# Classification of Daily Activities for the Elderly Using Wearable Sensors

**DOI:** 10.1155/2017/8934816

**Published:** 2017-11-26

**Authors:** Jian Liu, Jeehoon Sohn, Sukwon Kim

**Affiliations:** ^1^Division of Applied Science and Technology, Marshall University, Huntington, WV, USA; ^2^Department of Physical Education, College of Culture Convergence, Jeonju University, Jeonju, Republic of Korea; ^3^Department of Physical Education, College of Education, Chonbuk National University, Jeonju, Republic of Korea

## Abstract

Monitoring of activities of daily living (ADL) using wearable sensors can provide an objective indication of the activity levels or restrictions experienced by patients or elderly. The current study presented a two-sensor ADL classification method designed and tested specifically with elderly subjects. Ten healthy elderly were involved in a laboratory testing with 6 types of daily activities. Two inertial measurement units were attached to the thigh and the trunk of each subject. The results indicated an overall rate of misdetection being 2.8%. The findings of the current study can be used as the first step towards a more comprehensive activity monitoring technology specifically designed for the aging population.

## 1. Introduction

With advanced technology such as wireless communication and biomedical engineering, recording physiological and movement signals during daily activities was made possible [1, 2]. Isolating characteristics of body movement patterns during daily activities could provide functional status of the elderly [3, 4] or patients with motion impairments [5].

Traditional ADL (activities of daily living) assessment tools included self-reports, diaries, questionnaires, or subjective judgments by clinicians [6, 7]. But, these tools are typically retrospective and include personal opinions which can result in inaccurate decisions [6, 8]. In order to improve the reliability and validity of assessing daily activities, the use of wearable motion sensing technology has been adopted [1]. Simultaneously, long-term monitoring of activities of daily living was made possible through miniaturized motion sensors or wearable motion sensors [9–11].

There is a range of wearable sensors (e.g., accelerometers and gyroscopes) that are commonly used in monitoring human activities. Compared to the conventional lab-based measurement systems, wearable sensors have the general advantage of being portable, lightweight, cost-effective, and extremely suitable for long-term health monitoring in a daily living environment [1, 2, 10–12]. One of the application areas of wearable sensors is monitoring of activities of daily living (ADL), which can provide an objective indication of the activity levels or restrictions experienced by patients or elderly. Quantifying daily activities of the elderly collected by wearable sensors would provide meaningful data for clinical and research field in evaluating the level of functionality of the elderly [3, 4, 10]. A study [13] attempted to classify the daily activities of 26 young adults in the laboratory by using a single sensor attached to the waist and reported a sensitivity of 97.7% and a specificity of 98.7% over a date set of 1309 movements. However, in this experiment, comprehensive classification could not be achieved since only one sensor was used to measure accelerations. In the present study, in order to reflect the movement of the whole body and to achieve a more comprehensive classification of activities, two sensors in the trunk and thigh were used to find accelerations at different parts of the body.

A detailed assessment of ADL offers a promising approach to objectively evaluate the effectiveness of experimental manipulations or medical interventions, such as rehabilitation programs, surgeries, and medications [14]. Back in 1995, a study [15] performed an initial study to establish a starting point for ambulatory ADL monitoring via accelerometers. Recently, Zhang et al. [16] reported a new microcomputer-based portable physical activity measurement device (IDEEA), which is capable to detect 32 types of regular physical activity. Over the years, many methods have been proposed to classify ADL using accelerometers or gyroscopes. However, implementations particularly designed for the elderly population have been overlooked in the literature.

Therefore, the objective of the study was to design and evaluate a method to classify daily activities for the elderly based on a two-sensor system. It was expected that the proposed method could achieve satisfactory detection performance, as quantified by the rate of misdetection. The findings from the study would contribute to the understanding of activity characterization for the elderly population.

## 2. Methods

### 2.1. Detection Algorithm

**Figure 1:**
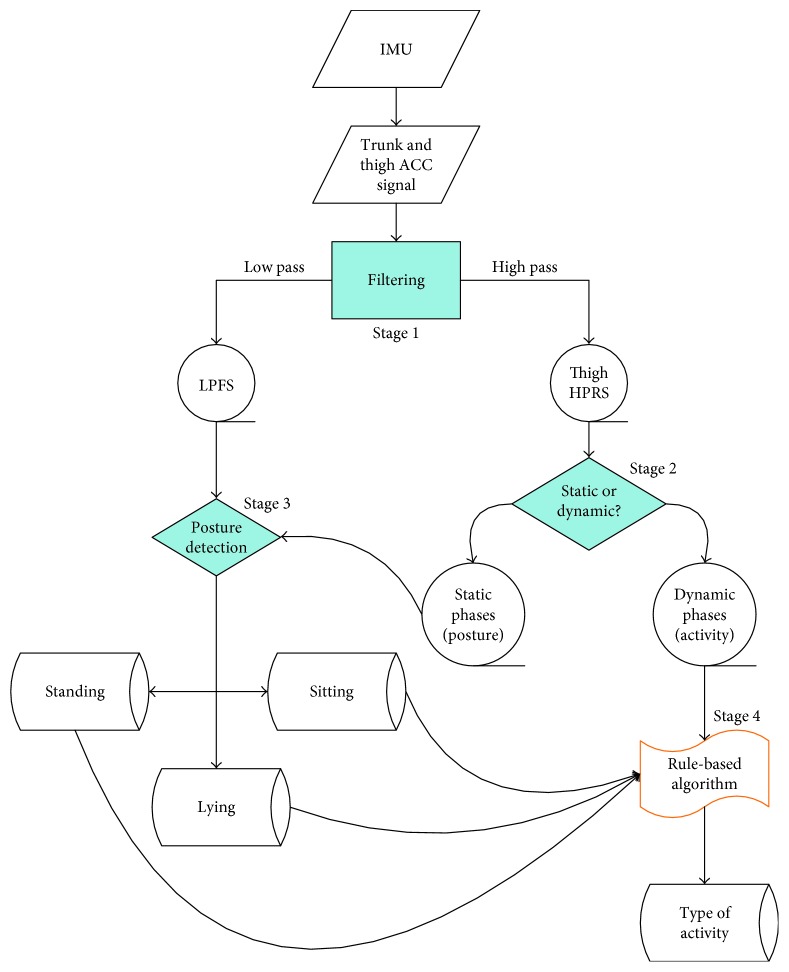
Illustration of ADL classification scheme.

The general principle of this ADL classification is to detect the body postures during static phases and then to recognize the types of dynamic activities between postures using a rule-based approach [17]. The types of dynamic activities can include various postural transitions, locomotor activities, and other motions. The schematic illustration of the ADL classification is shown in Figure 1. The entire processing was divided into four stages.

#### 2.1.1. Stage 1 Filtering

Both the trunk and thigh accelerometer signals were first low-pass filtered (Butterworth, 4th order, 0.5 Hz). The resulting signal (LPFS) was used in posture detection. The thigh accelerometer signal was further high-pass filtered, rectified, and smoothed [14]. The resulting signal (HPFS) was used to differentiate dynamic and static phases using the threshold technique.

#### 2.1.2. Stage 2 Static or Dynamic Phases

Differentiation between static (posture) and dynamic phases (postural transition and activity) was achieved by applying the threshold technique to the thigh HPFS signal. The rationale was that the more “dynamic” a motion is, the more “variable” the accelerometer signal will be, and it will contain more high-frequency components [14]. The threshold was empirically determined to be 0.04 g. The entire thigh HPRS signal was segmented by this threshold into multiple static and dynamic phases.

#### 2.1.3. Stage 3 Posture Detection

Posture detection was performed in static phases identified by stage 2. The thigh and chest LPFS signals were used to estimate the orientation of the thigh and trunk segments, respectively, using [18]

(1) $$\begin{array}{c} \Theta =\tan^{-1}\frac{\alpha_{z}}{\alpha_{x}}, \end{array}$$

where *a_z_* is the accelerometer signal in the longitudinal direction and *a_x_* is the signal in the frontal direction. The combination of the thigh and trunk segment orientations was used to identify the posture using a best-estimate approach [19]. Because the reference values for the healthy elderly population are not available in the literature, the orientation thresholds (Table 1) were empirically determined from the data collected in the current study.

#### 2.1.4. Stage 4 Activity Detection

The types of dynamic motion (activity or postural transition) for each dynamic session were determined by the posture types of the adjacent static phases. A rule-based algorithm specifies all the possible transitions between postures. Specifically, the transitional activity between “standing” is considered as “walking.” The activity between the posture “standing” and the posture “sitting” is considered as the “sitting down” activity. Dynamic level while sitting was used to determine the type of sitting. It should be noted that this rule-based scheme is not meant to be exhaustive and only considers those possible activities/activity transfers that are common for a typical older adult.

### 2.2. Laboratory Testing

Ten elderly participants (age = 75 ± 6 years, weight = 74.1 ± 9.1 kg, height = 174 ± 7.5 cm) were involved in a laboratory testing. They were required to be in generally good physical health. Informed consent was approved by the IRB committee at Virginia Tech and obtained from the participants prior to data collection.

One inertial measurement unit (Inertia-Link, MicroStrain, Inc., USA) was placed close to the sternum. The Inertia-Link is a miniature orientation sensor which is capable of measuring 3D orientation, 3D acceleration, and 3D angular velocity. With respect to the orientation performance, the Inertia-Link has an angular resolution of <0.1°, static accuracy of ±0.5°, and dynamic accuracy of ±2.0° RMS (MicroStrain, Inc., 2007). The dynamic ranges for the acceleration and angular velocity outputs are ±300°/s and 5 g, respectively (MicroStrain, Inc., 2007). Another inertial unit was placed on the right thigh (front side and mid-point) of the participant. WiTilt contains a miniature triaxial accelerometer (MMA7260Q, Freescale Semiconductor, USA), with selectable dynamic ranges of 1.5 g, 2 g, 4 g, and 6 g. The typical sensitivity is 800mv/g at 1.5 g (Freescale Semiconductor, 2005). The sampling rate for both units was set to be 100 Hz.

**Figure 2:**
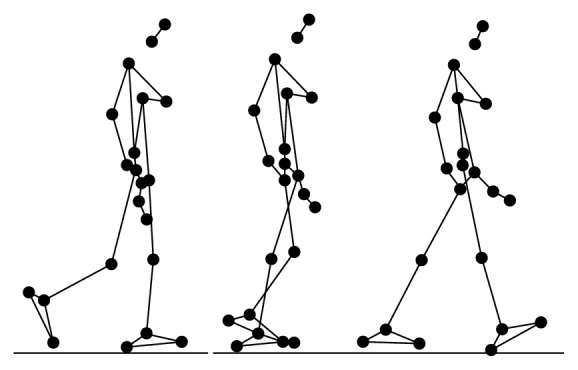
Illustration of normal walking activity (N).

**Figure 3:**
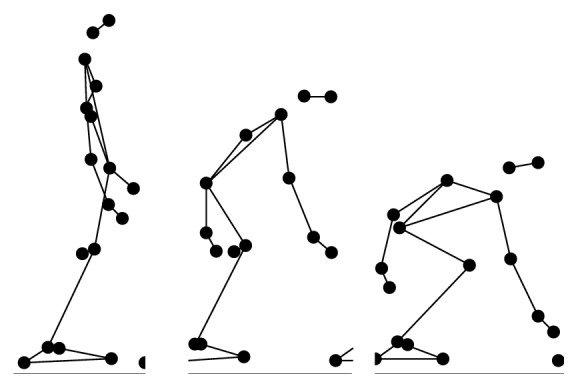
Illustration of bending over to pick up an object (BD).

**Figure 4:**
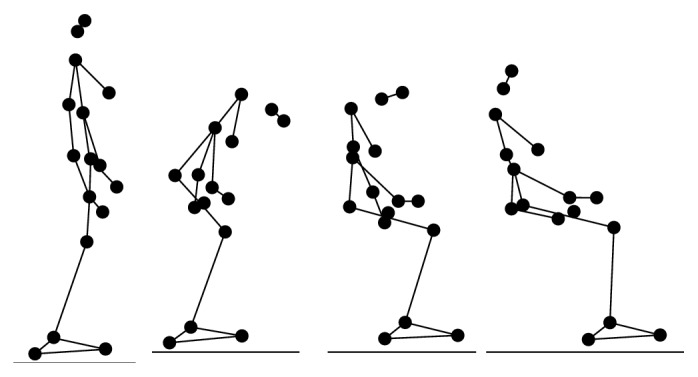
Illustration of sitting down on a regular chair (SN).

**Figure 5:**
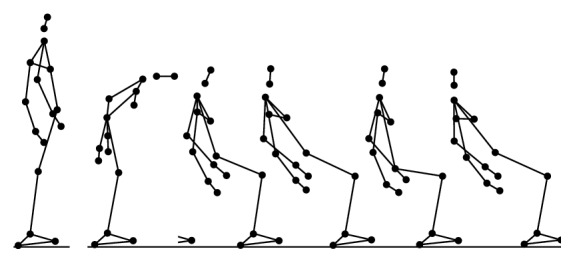
Illustration of sitting into a rocking chair (SR).

**Figure 6:**
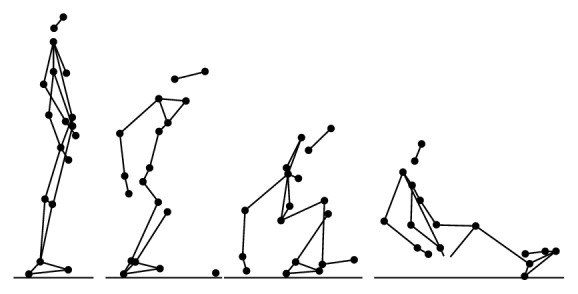
Illustration of sitting into a bucket seat (SB).

**Figure 7:**
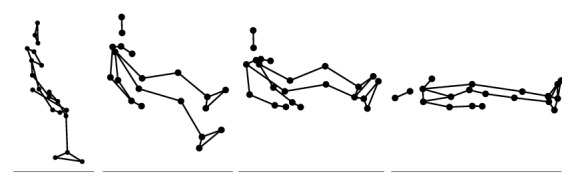
Illustration of lying down onto a medical bed (LD).

During the experiment, each participant performed 6 types of daily activities (Figures 2, 3, 4, 5, 6, and 7), including sitting down on a regular chair, sitting into a rocking chair, sitting into a bucket seat, lying down onto a medical bed, bending over to pick up an object from the ground, and walking. They were instructed to perform these activities as naturally as possible at their own pace. The presentation order of the daily activities was randomized using Latin square design. These activities were chosen for two reasons. First, they are representative of the activities that an older adult would perform on a daily basis. Second, several of these ADL (e.g., bending over and sitting down) have been considered challenging for an effective fall detection algorithm in the literature [6].

Data acquisition was performed by a custom-designed program in LabVIEW 8.2 (National Instruments, TX, USA). Receiver operating characteristic (ROC) analysis was performed to quantify the overall performance of the activity classification algorithm. The rate of misdetection was calculated to quantify the rate of incorrect classification for each type of activities. All the analyses were performed in MATLAB (R2007b, MathWorks, USA).

## 3. Results

**Figure 8:**
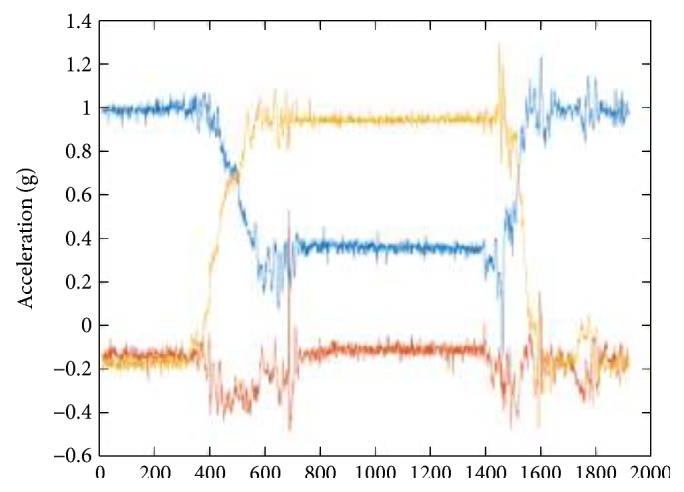
Raw 3D acceleration of the inertial unit on the thigh during a typical sitting down activity.

**Figure 9:**
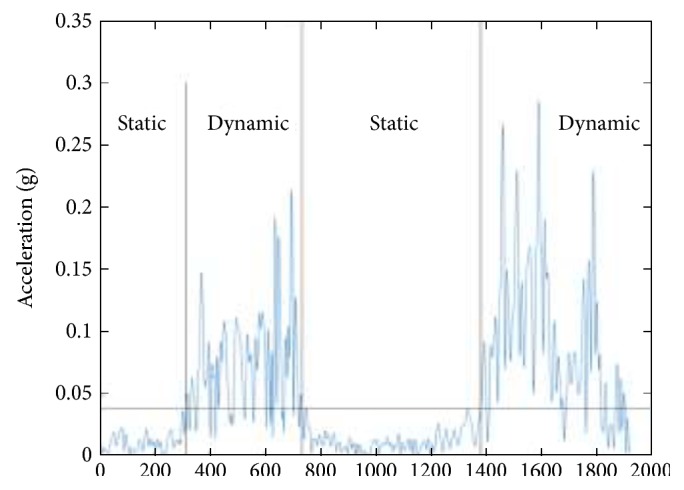
Resultant high-pass filtered signal (HPFS) of the thigh during a typical sitting down activity.

**Figure 10:**
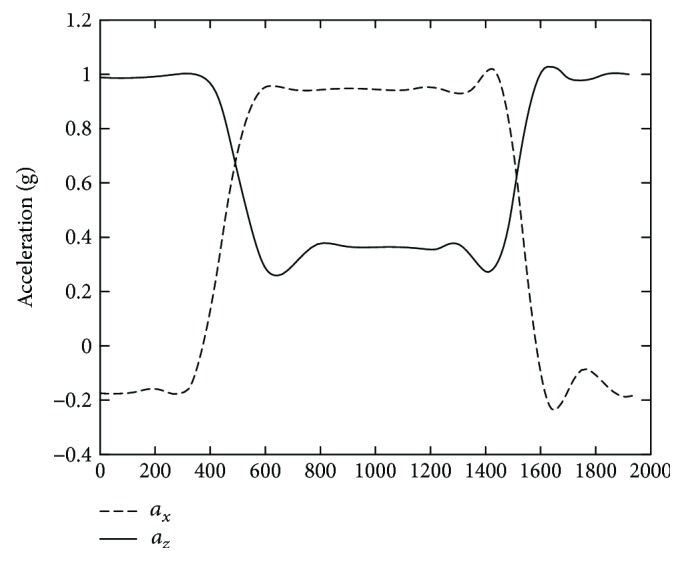
Low-pass filtered signal (LPFS) of the thigh during a typical sitting down activity.

**Figure 11:**
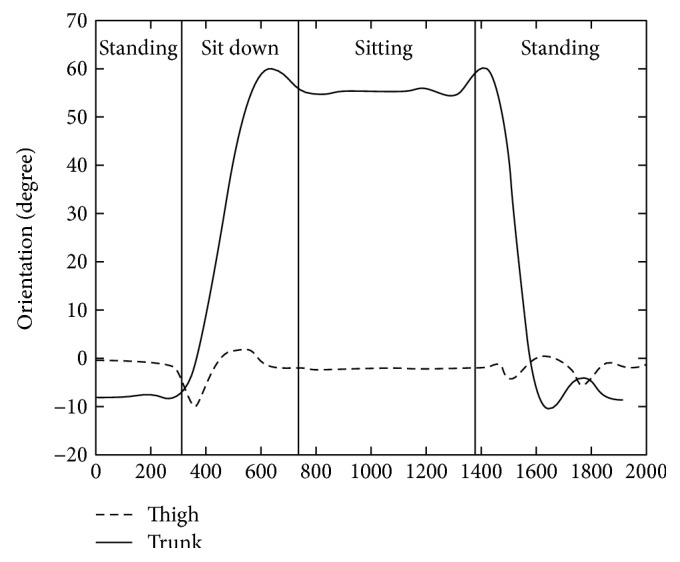
Sagittal orientation of the thigh and trunk during a typical sitting down activity.

The results of a typical sitting down activity trial is illustrated in Figures 8, 9, 10, and 11.  Figure 8 shows the raw 3D acceleration data measured from the inertial unit on the thigh segment.  Figure 9 shows the HPFS with static and dynamic motion ranges detected by the 0.04 g threshold.  Figure 10 shows the vertical acceleration (*a_z_*) and the horizontal acceleration (*a_x_*) of the LPFS.  Figure 11 shows the sagittal orientation of the thigh and trunk, along with the posture and activity detected.

Total of one hundred and seventy-nine (179) ADL trials were collected and subject to the ADL classification algorithm. As shown in Table 2, all trials (89 trials) for the three types of ADL (bending over (29 trials), lying down (30 trials), and normal walking (30 trials)) were classified correctly without any misdetection. One trial of sitting on a normal chair was incorrectly classified for bending over to pick up an object from the ground. Four other trials were not recognized by the detection algorithm and were not classified. Of the five unsuccessful trials, the three trials resulted from sensor measurement errors and the two other trials were due to the difficulty to detect a clear static posture at the end of the trial by the algorithm. Overall, the ADL in the current study were able to be classified with only 2.8% misdetection rate.

## 4. Discussions and Conclusions

Even though there are several tools available to evaluate the physical activity of the elderly, these methods have limited reliability or validity and unsuitable for longer time periods of measurement [9]. The use of wearable motion sensing technology for studying human motion has overcome the limitations. Monitoring of daily activities has been investigated with younger and middle-aged adults by many researchers [4, 10, 13]. The current study presented a method specifically for the elderly population.

The findings from the current study showed an outstanding classification performance of the ADL classification algorithm. The misdetection rate was found to be 2.8%. A portion (two out of five) of these misdetections was due to the nonuniform movement patterns. As most real-world activities are nonuniform, it is advised to deploy more sophisticated ADL classification method in the future development for practical consideration.

He et al. [20] reported the 527 correct classifications out of 550 trials (an accuracy of 95.82%) using a Hidden Markov Model as compared to the accuracy of 97.2% of the present study. In the previous study, a subject performed 11 different activity series resulted from 8 different activity combinations, whereas, in the present study, a subject performed 6 different activities such as SN, SR, SB, LD, BD, and N. The activity levels of the previous study [20] may have introduced a larger portion of nonuniform motions compared to the present study leading to higher misdetection rate [20, 21]. The performance from the present study compared favorably to those of the other algorithms in the literature [19, 20, 22, 23]. For example, the best-estimate threshold approach [19], which was the basis for the current ADL algorithm, achieved an overall accuracy ranging from 84% to 97% for different types of activities. Most recently, Karantonis et al. [23] implemented a real-time ADL monitor system and observed an overall misdetection rate of 10.2%.

It should be acknowledged that many simplifications existed both in the algorithm and in the ADL testing protocol employed in the current study compared to a study [20]. In terms of the algorithm, one major simplification was to classify walking and bending over based on the length of the time interval between consecutive standing postures. In terms of the testing protocol, one major simplification was that the ADL were not performed continuously. Instead, participants were asked to stand still at the beginning and the end of each trial. In addition, as is true for most of the similar studies, the types of ADL in the current study were, by no means, exhaustive or representative to all the ADL that one would experience in daily life [21]. It is advised to adopt less controlled activity protocol in a seminaturalistic environment in the future [21, 22, 24]. One idea would be to have the participants move freely in a semiliving environment and to have their motion features monitored continuously without interruption for a period of time. In addition, the study is limited to elderly participants. Future studies involving different age groups would allow investigation in the aging effect on activity detection parameters.

In conclusion, the current study successfully designed and implemented a two-sensor daily activity detection algorithm with elderly subjects. This is the first step towards a more comprehensive activity monitoring technology specifically designed for the aging population. The study findings contributed to the understanding of activity motion features of the aging population.

## Figures and Tables

**Table 1:** Orientation threshold for posture detection.

Segment	Posture					
	Sitting		Standing		Lying	
	Range	Reference	Range	Reference	Range	Reference
Trunk	±60°	Vertical	±20°	Vertical	−30°~45°	Horizontal
Thigh	±60°	Horizontal	±30°	Vertical	±30°	Horizontal

**Table 2:** Summary of classifications of one hundred seventy-nine (179) trials.

Activities	Number of trials	Number of misdetection
BD	29	0
LD	30	0
SB	30	0
SN	30	2
SR	30	3
N	30	0

BD: bending over to pick up an object from the ground; LD: lying down onto a medical bed; SB: sitting into a bucket seat; SN: sitting on a regular chair; SR: sitting into a rocking chair; N: normal walking
